# Supplementary material for: A gross margin analysis for Nguni cattle farmers in Limpopo Province, South Africa
Source: PLoS One. 2021 Jun 24;16(6):e0253657. doi: 10.1371/journal.pone.0253657 (PMC8224916; doi:10.1371/journal.pone.0253657)
Supplement: Attachment — Submitted filename: PlosOne rebattal.docx [file pone.0253657.s002.docx]

Response to Reviewers

**Data availability on request**

The data for this manuscript is available on request. The university of Limpopo requires that if data is to be shared from student’s thesis, such as is the case with our article, then a request can be sent to the corresponding author upon receipt of which the corresponding author approaches that University of Limpopo’s Research Ethical Committee which ensures that the data are sufficiently de-identified after which the corresponding author can submit data to the requestor.

| **Reviewer comment** | **Authors’ response** |
| --- | --- |
| **Reviewer #1** |  |
| There is no literature review in the article; it should be written in 2-3 pages | Literature added. |
| The content of the chapter "Objectives" is rather belonging to the introduction | The chapter “Objectives” removed. Combined with preceding chapter |
| There are no limitations described. | Limitations added |
| In the conclusions, I couldn't find what is the relevance of this research, what it can add to the existing knowledge and what are the consequences for the policy making and practice. | Conclusion revised for policy relevance. |
| There are too many subchapters in the text | One chapter removed. However, we feel that the remaining chapters are necessary for better understanding. |
| **Reviewer #2.** |  |
| On the other hand, the number of cited literature is not that high in the present form | Literature added. |
